# Supplementary material for: Inhibition of polar actin assembly by astral microtubules is required for cytokinesis
Source: Nat Commun. 2021 Apr 23;12:2409. doi: 10.1038/s41467-021-22677-0 (PMC8065111; doi:10.1038/s41467-021-22677-0)
Supplement: Supplementary file 1 — Supplementary Information [file 41467_2021_22677_MOESM1_ESM.pdf]

Supplementary Figure 1.

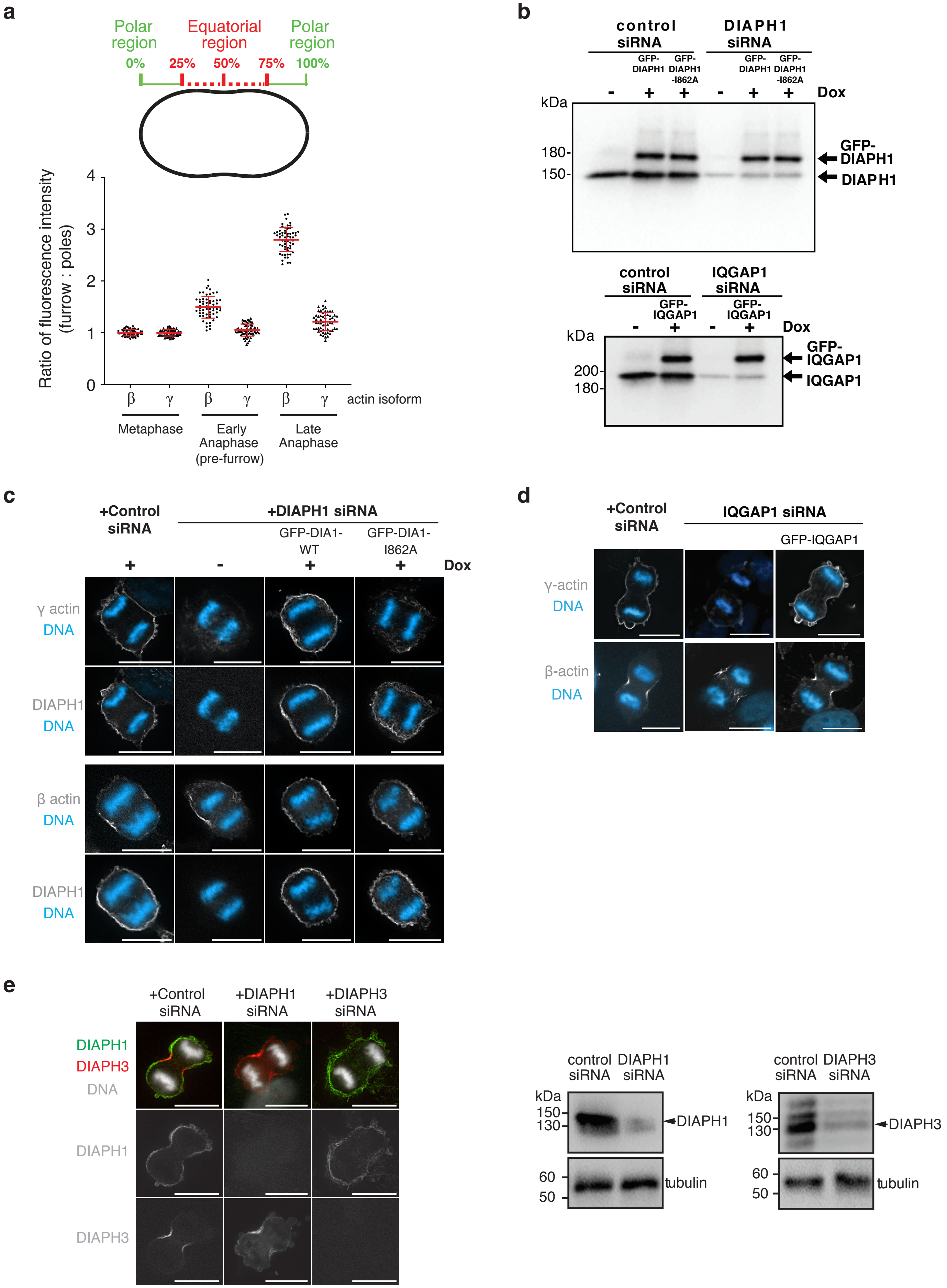

Supplementary Figure 1.

Characterization of actin isoforms and DIAPH1, 3 and IQGAP1 during anaphase.

(**a**) Comparative distribution of β- and γ-actin localization between the equatorial and polar regions in cytokinetic cells. n = 60 cells examined. Bars = average, whiskers = ± SD. (**b**) Western blots of HeLa cell lysates from stable cell lines expressing GFP-DIAPH1, GFP-DAIPH1-I862A or GFP-IQGAP1 treated with control siRNA or siRNA targeting DIAPH1 or IQGAP1. (**c**) Localization of DIAPH1, β- and γ-actin in the presence or absence of endogenous DIAPH1, the presence of only GFP-DIAPH1 or GFP-DIAPH1-I862A in pre-furrowing anaphase cells. (**d**) Localization of β- and γ-actin in HeLa cells in the presence or absence of endogenous IQGAP1 or the presence of only GFP-IQGAP1. (**e**) DIAPH1 and DIAPH3 do not influence each other's cellular localization. In the left panels, HeLa cells were treated with control, DIAPH1 or DIAPH3 siRNA then fixed and probed with antibodies recognizing DIAPH1 and 3. In the right hand panels, western blots of lysates from HeLa cells treated with either control, DIAPH1 or DIAPH3 siRNA and probed with DIAPH 1 and 3 antibodies. All scale bars 10μm, DNA visualized by DAPI staining. Uncropped blots are shown in Supplementary Fig. 6.

Supplementary Figure 2.

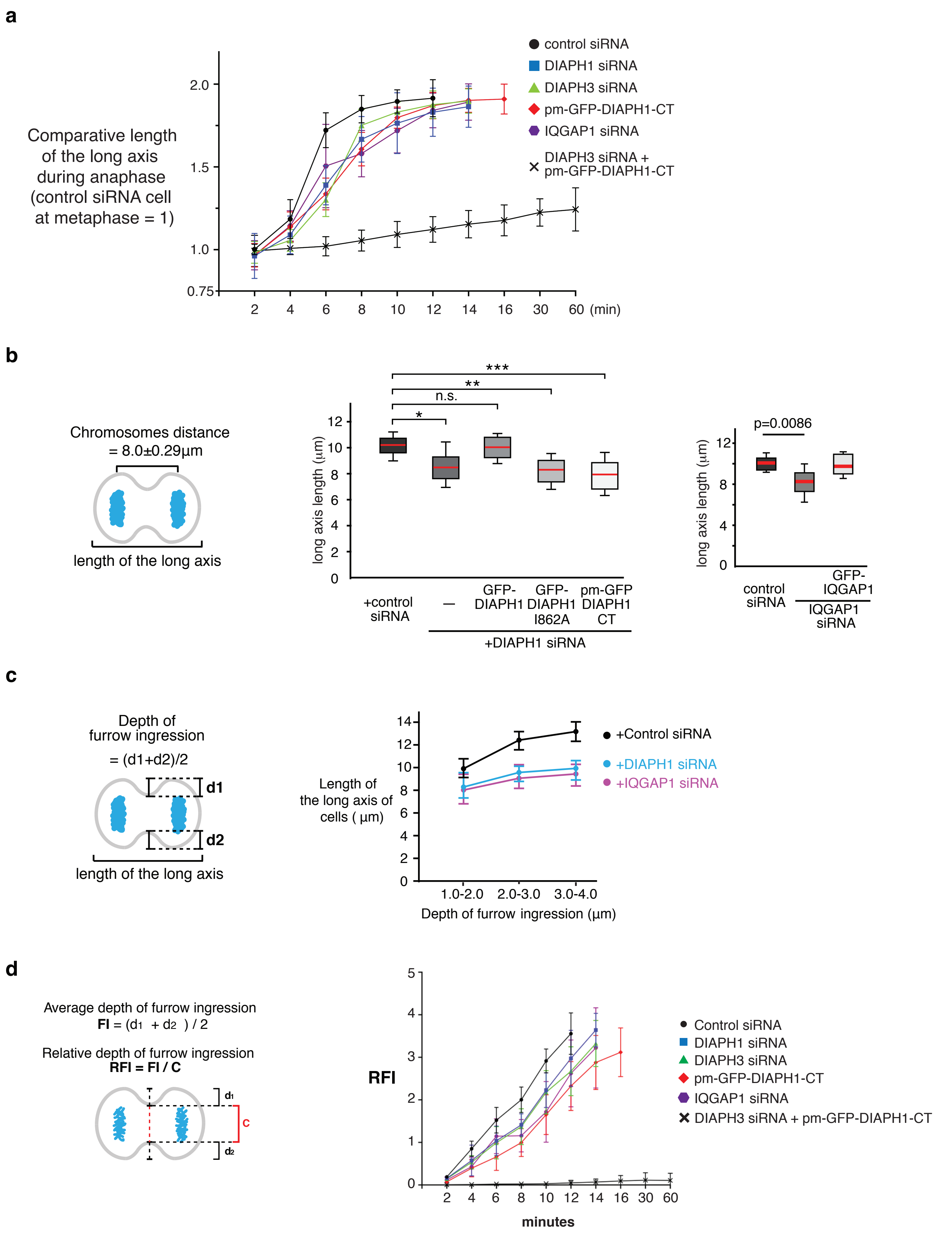

Supplementary Figure 2.

Perturbing distinct actin networks during anaphase affects cell elongation and furrow ingression. (**a**) The long axis of siRNA treated cells was measured as the cells progressed from metaphase through anaphase and compared to the elongation of control siRNA treated cells. The rate of cell elongation was determined between 2 and 8 mins post metaphase and is plotted in Fig 1f. n = 10 cells examined in each live-cell imaging group. Whiskers = $\pm$ SD. (**b**) The long axis of fixed siRNA treated anaphase cells (with an inter chromosomal distance was 8μm) is shown. Schematic describing how the measurements were made. *p = 0.005, **p = 0.004, ***p = 0.003 to control using non-parametric two-tailed Mann-Whitney *t* tests for 3 experimental repeats. n = 100 cells examined over 3 independent experiments. Bars = average, boxes = 25-75 percentile, whiskers = data extreme. (**c**) Long axis of fixed siRNA treated anaphase cells at three different average furrow ingression depths. Schematic describes how the measurements were made. n=100 cells examined over 3 independent experiments at each stage for each condition. Dots = average, whiskers = $\pm$ SD. (**d**) Schematic outlining how the relative depth of furrow ingression was determined and compared between conditions. The depth of each side of the furrow was measured, then averaged before being divided by the width the intercellular collar between the 2 forming daughter cells. Furrow depth parameters were measured over time to compare the relative furrow ingression dynamics in different conditions. n = 5 cells examined in each live-cell imaging group. Whiskers = $\pm$ SD.

Supplementary Figure 3

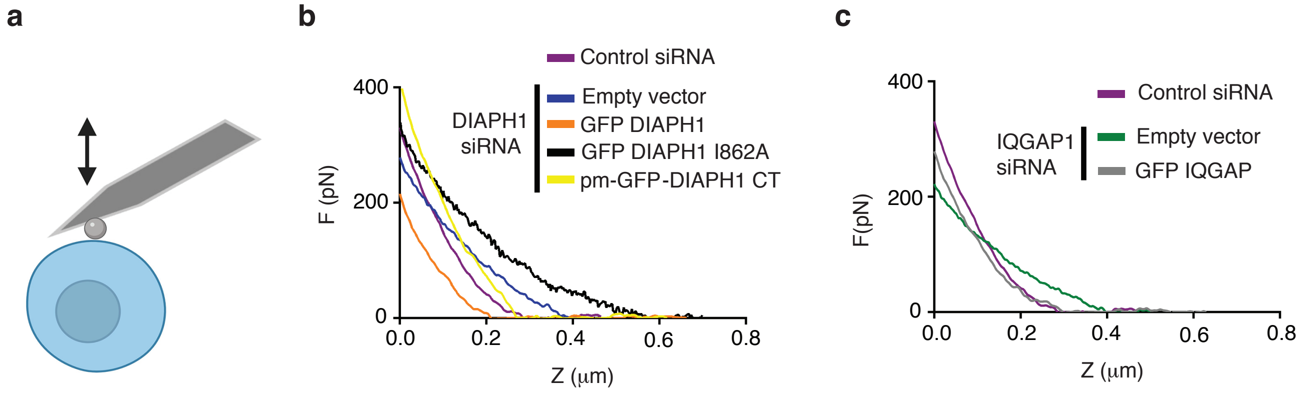

d

	control cells	control siRNA	DIAPH1 siRNA				IQGAP1 siRNA	
				+GFP DIAPH1	+GFP DIAPH1 I862A	+pm GFP DIAPH1 CT		
Mean	2.068	2.116	0.661	1.251	0.256	2.293	0.625	1.296
S.D.	0.756	0.728	0.111	0.231	0.067	0.542	0.090	0.237
number of cells	29	29	29	27	29	29	27	28

Supplementary Figure 3
Metaphase Cell Surface Stiffness measured by Atomic Force Microscopy (AFM).
(a) Schematic representation of the technique used to measure cortical stiffness.
(b) Representative force-indentation curves for cells transfected with control siRNA or endogenous DIAPH1 siRNA. DIAPH1-silenced cells were also transfected with empty vector, or plasmids encoding GFP-DIAPH1, GFP-DIAPH-I862A or pm-GFP-DIAPH1-CT.
(c) Representative force-indentation curves for cells transfected with control siRNA or IQGAP1 siRNA. IQGAP1-deficient cells were also transfected with empty vector or a plasmid encoding GFP IQGAP1.
(d) Average Young's modulus values, in kPa, measuring plasma membrane tension at metaphase in HeLa cell lines expressing different levels of DIAPH1.
Abbreviations: F, force. pN, picoNewtons. Z, depth of indentation.

Supplementary Figure 4.

a

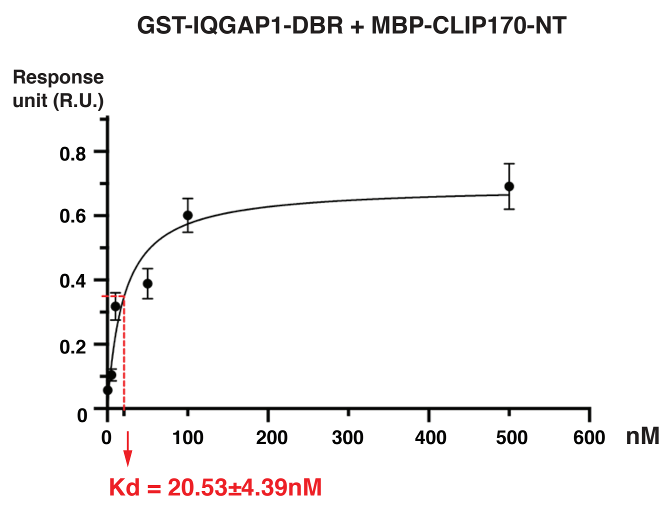

b

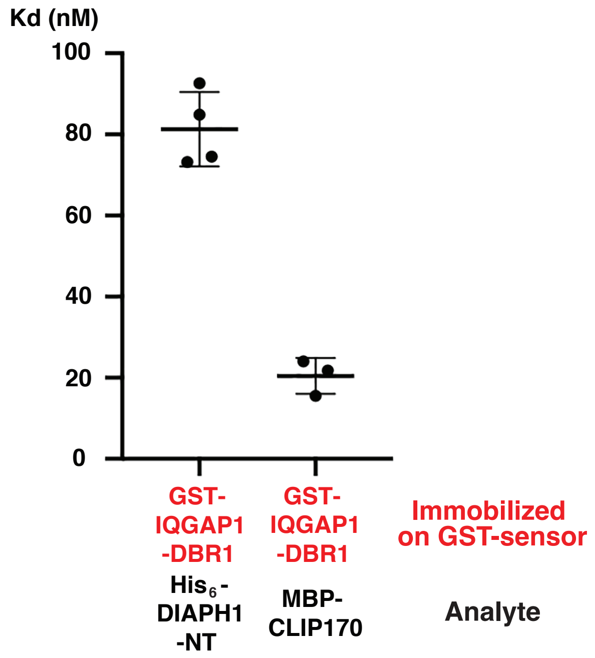

c

**Mean Kd (nM)**	20.5
**Kd** of each test	24.1<br>15.6<br>21.8
**±SD**	4.392
**±SEM**	2.536

Supplementary Figure 4.
IQGAP1 has a four-fold greater affinity for CLIP170 than DIAPH1.
(a) Representative binding curve measured using Biolayer Interferometry for the interaction between GST-IQGAP1-DBR and MBP-CLIP170. n = 3 independent reactions analyzed under each concentration condition. Whiskers = + SD.
(b) Dissociation constants plotted from 3 individual experiments between the protein pairs GST-IQGAP1-DBR with His_6-DIAPH1-NT and GST-IQGAP1-DBR with MBP-CLIP170. Each data point represents the average Kd from 3 tests. n = 4 (GST-IQGAP1-DBR with His_6-DIAPH1-NT), n = 3 (GST-IQGAP1-DBR with MBP-CLIP170). Bars denote the average, whiskers = + SD.
(c) Individual experimental values for the interaction of GST-IQGAP1-DBR with MBP-CLIP170. Data for the interaction between GST-IQGAP1-DBR and His_6-DIAPH1-NT is described in Chen et al., 2020.

Supplementary Figure 5.

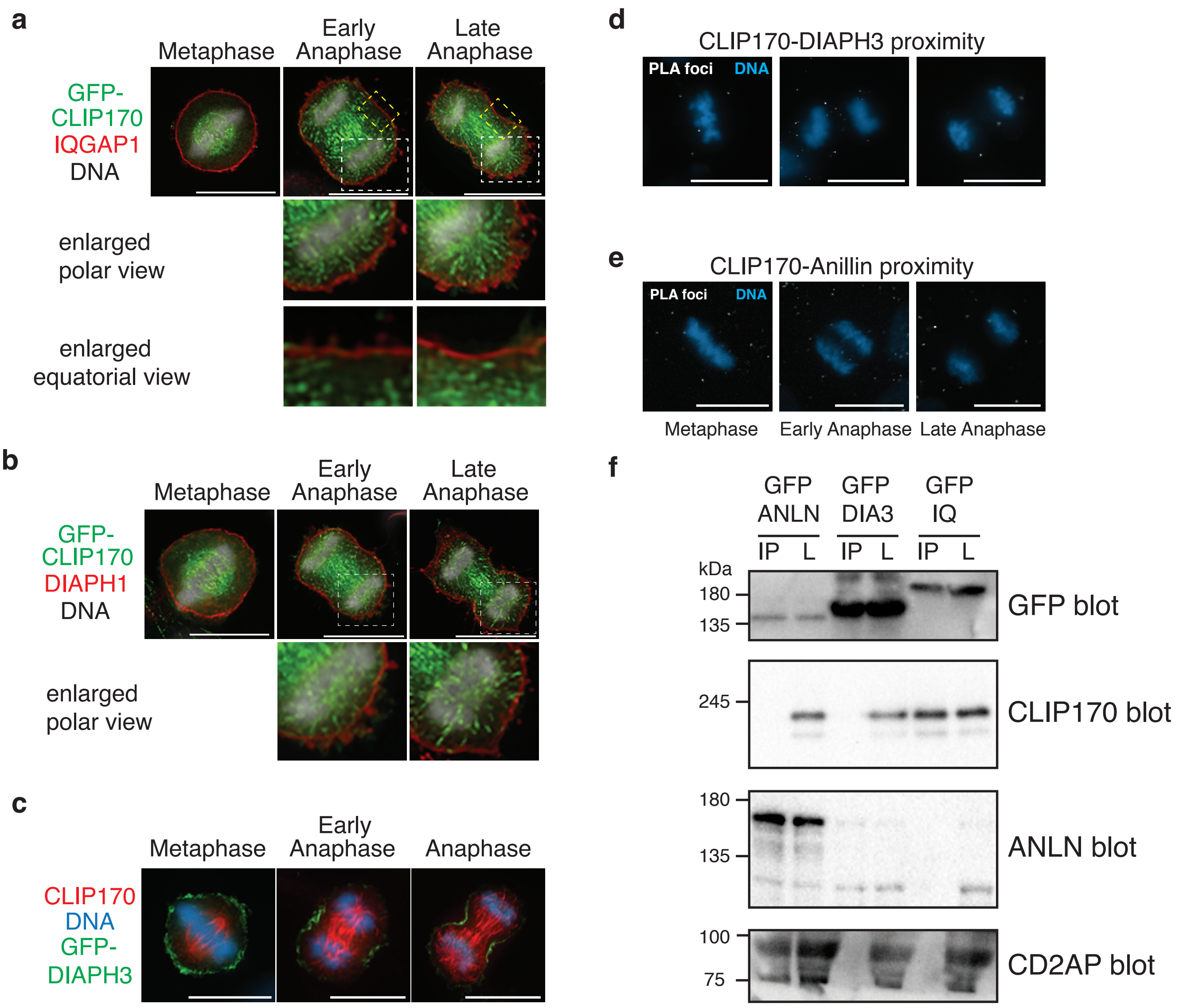

Supplementary Figure 5.
During anaphase CLIP170 is trafficked toward the pole and lacks proximity to equatorial cytokinetic actomyosin ring regulators.
GFP-CLIP170 expressing HeLa cells fixed and probed with **(a)** IQGAP1 and **(b)** DIAPH1.
(c) GFP-DIAPH3 expressing HeLa cells fixed and probed with a CLIP170 antibody.
(d) Proximity ligation assay (PLA) for CLIP170 and DIAPH3 in HeLa cells expressing GFP-DIAPH3 probed with the anti GFP and CLIP170 antibodies.
(e) Proximity ligation assay (PLA) for CLIP170 and anillin in HeLa cells expressing GFP-CLIP170 probed with the anti-GFP and anillin antibodies.
(f) GFP tagged proteins were immunoprecipitated from stable HeLa cells lines expressing GFP-anillin (GFP-ANLN), GFP-DIAPH3 (GFP-DIA3) and GFP-IQGAP1 (GFP-IQ). Immunoprecipitates were analyzed by western blotting using antibodies recognizing GFP, CLIP170, anillin (ANLN) and CD2AP a known anillin interactor.

Supplememntary Figure 6.

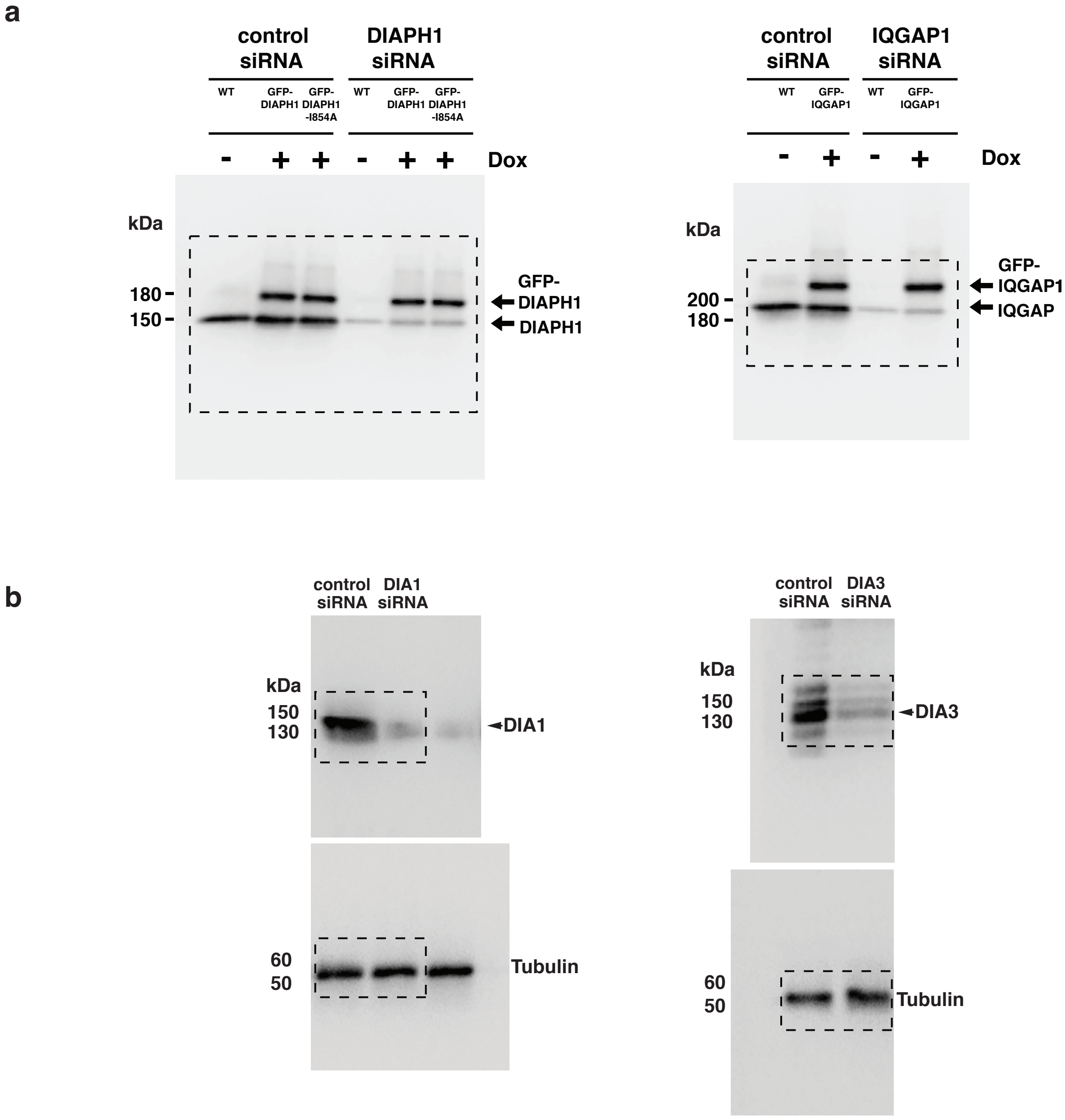

Supplementary Figure 6. (a) uncropped blots associated with Supplementary Fgiure 1b
(b) uncropped blots associated with Supplementary Fgiure 1e

Supplementary Figure 7.

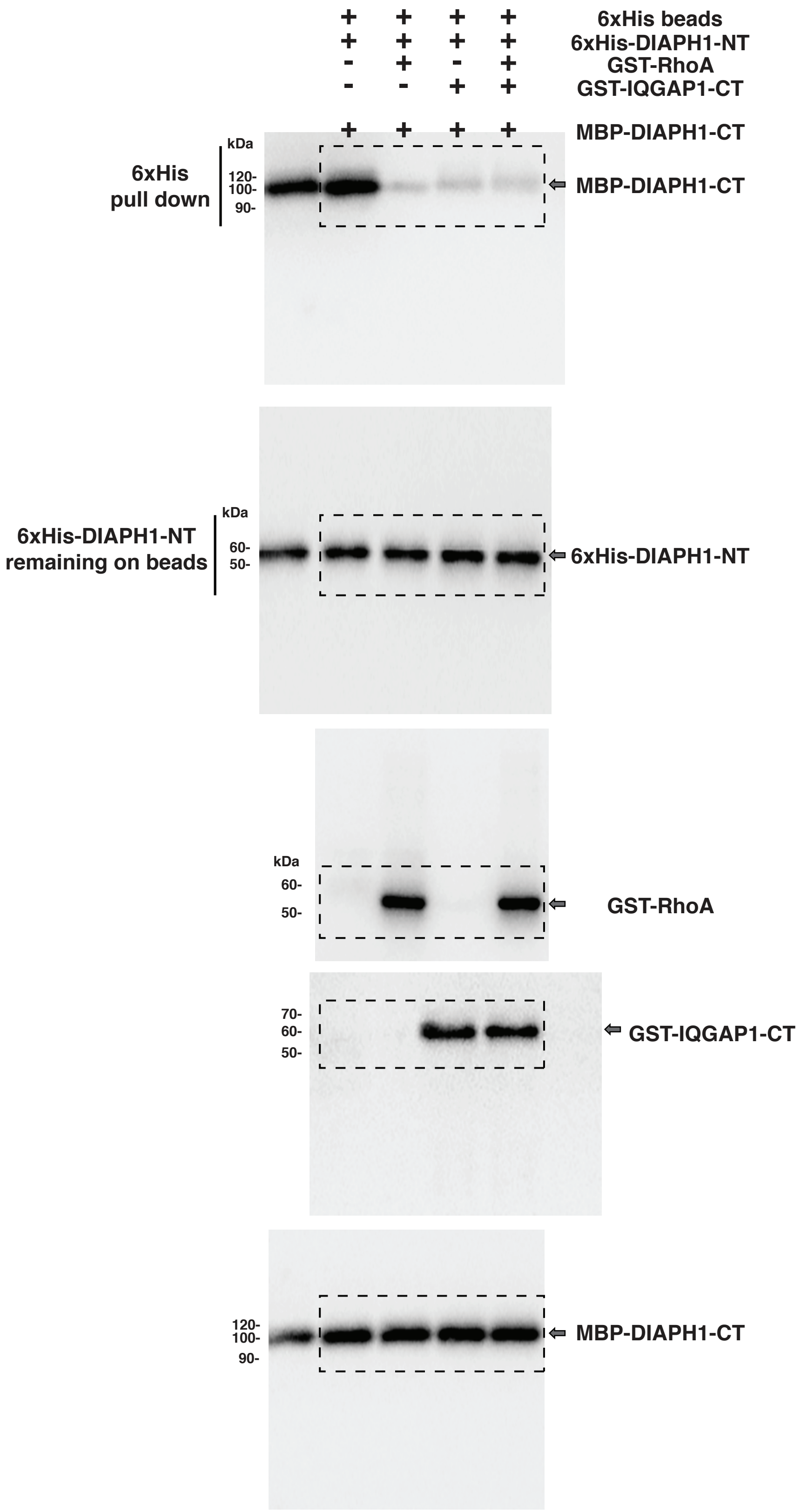

Supplementary Figure 7. Uncropped blots associated with Figure 5

Supplemetary Figure 8.

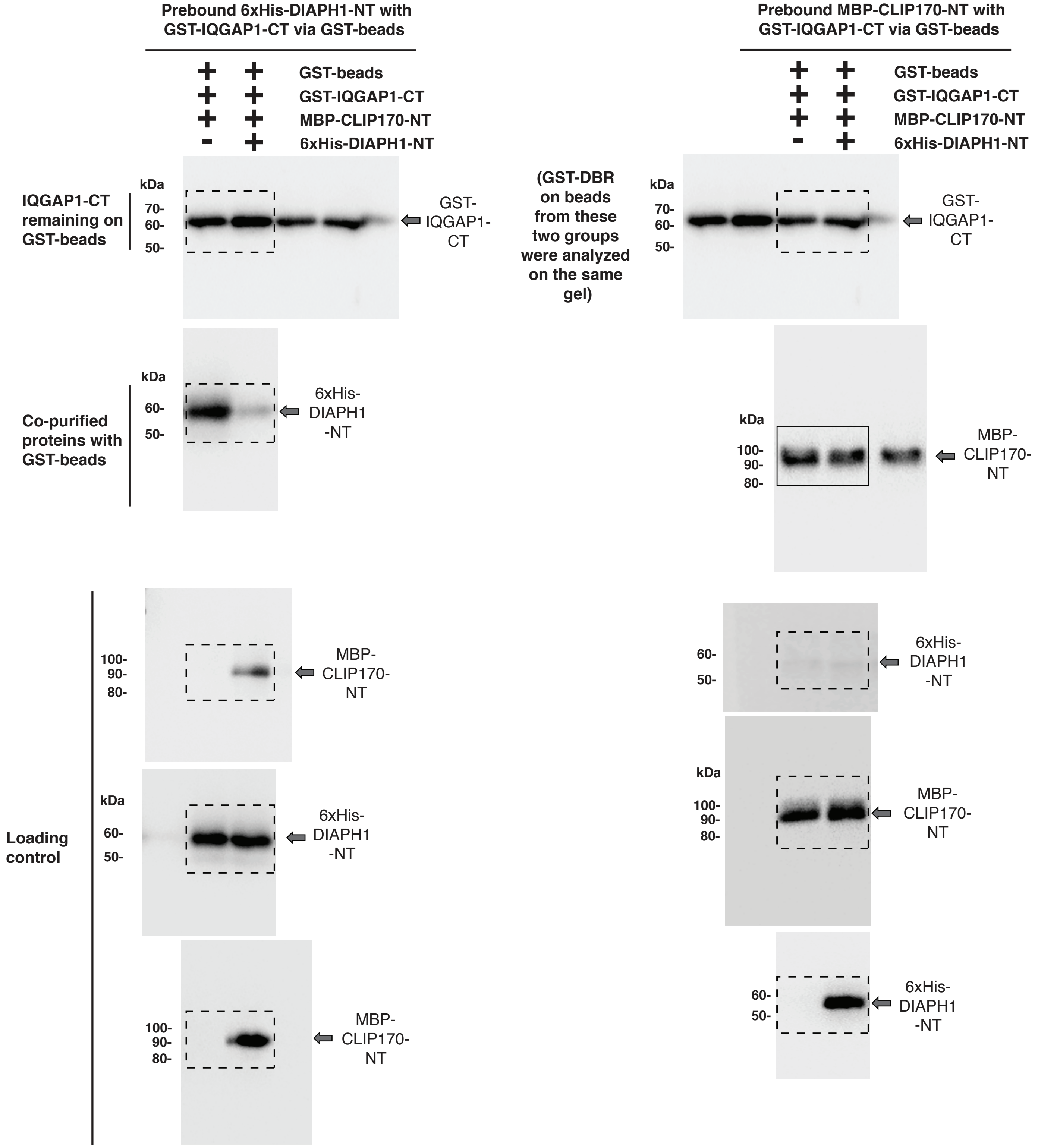

Supplemetary Figure 8. Uncropped blots associated with Figure 6.

Supplementary Table 1

DIAPH1_5’_fwd	5’-ATGGAGCCGCCCGGCGGGA
DIAPH1_583aa_fwd	5’-TCTGGCACTATTATTCCACCAC
DIAPH1_3’_rev	5’-TTAGCTTGCACGGCCAACCAA
DIAPH1_570aa_fwd<br>*(used to generate 5’overlap on DIAPH1-CT to anneal with 3’-DIAPH1-NT)*	5’-<br>GCTCCTGTTCCCCCTGCCCCTCCTTTACCTGGTGACTC<br>TGGCACTATTATTCCA
DIAPH1_580aa_rev<br>*(used to generate 3’ overlap on DIAPH1-NT to anneal with 5’-DIAPH1-CT)*	5’-<br>AGGTAAAGGAGGGGCAGGGGGAACAGGAGCACGACT
DIAPH1-I862A_5’_fwd	5’-GCCCAGAATCTCTCAATCTTTTTGGGTTCC
DIAPH1-I862A_3’_rev	5’-GGAACCCAAAAAGATTGAGAGATTCTGGGC
IQGAP1_5’_fwd	5’-ATGTCCGCCGCAGACGAGGTT
IQGAP1_1500aa_fwd_<br>NotI	5’-CCAGTCACTATGGCGGCCGCTTCATGGACTCT-3’
IQGAP1_1657aa_3’_rev_<br>HindIII	5’-GCTTATCATCGATAAGCTTTTACTTCCCGTAG-3’
CLIP170_5’_fwd_NotI	5’-<br>CCAGTCACTATGGCGGCCGCATGAGTATGCTGAAACC<br>CAGCGGG
CLIP170_350aa_rev_<br>EcoRI	5’-<br>CATTGCCATACGGAATTCAGTGGTGCCCGAGATCTTG
CLIP170_500aa_fwd_<br>NotI	5’-<br>CCAGTCACTATGGCGGCCGCATGGAACTAGAAAAGGA<br>CCTAGCG
CLIP170_1320aa_3’_rev_<br>PstI	5’-<br>CACTATGGTCGACCTGCAGTCAGAAGGTCTCATCGTC<br>GTT

Supplementary Table 2

siRNA used in the study	
DIAPH1 siRNA duplexes *(against 3'UTR)* IDT # hs.Ri.DIAPH1.13.2	5'-AAUAGCUGGGGCUUGUAAACCUTT-3'<br>5'-GUUUCAAUGCUUUAUUAACAGUUGGAA-3'
DIAPH3 siRNA duplexes IDT # hs.Ri.DIAPH3.13.1	5'-AACCUGAAAUCAGCAUGAGAAGATT-3'<br>5'-AAUCUUCUCAUGCUGAUUUCAGGUUUA-3'
IQGAP1 siRNA duplexes *(against 3'UTR)* IDT # hs.Ri.IQGAP1.13.1	5'-ACAUUUACCAUGAAUUUACUUCCTC-3'<br>3'-GAGGAAGUAAAUUCAUGGUAAAUGUCA-3'
Negative control DsiRNA (NC1)	IDT Cat No. 51-01-14-04
