# Supplementary material for: Inhibition of polar actin assembly by astral microtubules is required for cytokinesis
Source: Nat Commun. 2021 Apr 23;12:2409. doi: 10.1038/s41467-021-22677-0 (PMC8065111; doi:10.1038/s41467-021-22677-0)
Supplement: Supplementary file 3 — Description of Additional Supplementary Files [file 41467_2021_22677_MOESM3_ESM.pdf]

Description of Additional Supplementary Files

File Name: Supplementary Movie 1.
Description: Phase contrast time-lapse movie of a HeLa treated with a control siRNA cell going through mitosis.

File Name: Supplementary Movie 2.
Description: Phase contrast time-lapse movie of a HeLa treated with a DIAPH1 siRNA cell going through anaphase.

File Name: Supplementary Movie 3.
Description: Phase contrast time-lapse movie of a HeLa expressing a constitutively active DIAPH1 cell going through anaphase.

File Name: Supplementary Movie 4.
Description: Phase contrast time-lapse movie of a HeLa treated with a DIAPH3 siRNA cell going through anaphase.

File Name: Supplementary Movie 5.
Description: Phase contrast time-lapse movie of a HeLa expressing a constitutively active DIAPH1 treated with a DIAPH3 siRNA cell going through anaphase.

Description: File Name: Supplementary Movie 6.
Time-lapse movie of a HeLa cell expressing GFP-CLIP170 going through cytokinesis.
