# Supplementary material for: Inhibition of polar actin assembly by astral microtubules is required for cytokinesis
Source: Nat Commun. 2021 Apr 23;12:2409. doi: 10.1038/s41467-021-22677-0 (PMC8065111; doi:10.1038/s41467-021-22677-0)
Supplement: Supplementary file 10 — Reporting Summary [file 41467_2021_22677_MOESM10_ESM.pdf]

nature research

Corresponding author(s): Andrew Wilde

Last updated by author(s): Feb 25, 2021

Reporting Summary

Nature Research wishes to improve the reproducibility of the work that we publish. This form provides structure for consistency and transparency in reporting. For further information on Nature Research policies, see our Editorial Policies and the Editorial Policy Checklist.

Statistics

For all statistical analyses, confirm that the following items are present in the figure legend, table legend, main text, or Methods section.

n/a	Confirmed	
☐	☒	The exact sample size (*n*) for each experimental group/condition, given as a discrete number and unit of measurement
☐	☒	A statement on whether measurements were taken from distinct samples or whether the same sample was measured repeatedly
☐	☒	The statistical test(s) used AND whether they are one- or two-sided<br>*Only common tests should be described solely by name; describe more complex techniques in the Methods section.*
☒	☐	A description of all covariates tested
☒	☐	A description of any assumptions or corrections, such as tests of normality and adjustment for multiple comparisons
☐	☒	A full description of the statistical parameters including central tendency (e.g. means) or other basic estimates (e.g. regression coefficient) AND variation (e.g. standard deviation) or associated estimates of uncertainty (e.g. confidence intervals)
☐	☒	For null hypothesis testing, the test statistic (e.g. *F*, *t*, *r*) with confidence intervals, effect sizes, degrees of freedom and *P* value noted<br>*Give P values as exact values whenever suitable.*
☒	☐	For Bayesian analysis, information on the choice of priors and Markov chain Monte Carlo settings
☒	☐	For hierarchical and complex designs, identification of the appropriate level for tests and full reporting of outcomes
☒	☐	Estimates of effect sizes (e.g. Cohen's *d*, Pearson's *r*), indicating how they were calculated

Our web collection on statistics for biologists contains articles on many of the points above.

Software and code

Policy information about availability of computer code

Data collection	Not applicable
Data analysis	GraphPad Prism 8, version 8.2.0 (GraphPad Software Inc), Metamorph v7.7.0.0 (Molecular Devices), AutoQuant X3,X3.1.1 (Media Cybernetics), Octet Software, v10.0 (ForteBio), Image Lab Software, v6.0.0 (BioRad Inc.), Adobe Photoshop CC 2019 (Adobe Inc), Adobe Illustrator CC 2019 (Adobe Inc), Adobe Reader 2019 (Adobe Inc) Microsoft Word 2019, Micrososft Excel 2019

For manuscripts utilizing custom algorithms or software that are central to the research but not yet described in published literature, software must be made available to editors and reviewers. We strongly encourage code deposition in a community repository (e.g. GitHub). See the Nature Research guidelines for submitting code & software for further information.

Data

Policy information about availability of data

All manuscripts must include a data availability statement. This statement should provide the following information, where applicable:
- Accession codes, unique identifiers, or web links for publicly available datasets
- A list of figures that have associated raw data
- A description of any restrictions on data availability

All data is available in the main text or the supplementary materials. Any plasmids and cell lines generated during and/or analysed during the current study are available from the corresponding author on reasonable request.

Field-specific reporting

Please select the one below that is the best fit for your research. If you are not sure, read the appropriate sections before making your selection.

- [x] Life sciences
- [ ] Behavioural & social sciences
- [ ] Ecological, evolutionary & environmental sciences

For a reference copy of the document with all sections, see nature.com/documents/nr-reporting-summary-flat.pdf

Life sciences study design

All studies must disclose on these points even when the disclosure is negative.

Sample size	(1). For the quantification of the numbers of multi-nucleate cells, each data point collected presents the percentage of multi-nucleate cells in a population of 30 cells within an optic field of the ×60/1.4 NA oil-immersion objective lens. At least 6 random fields were observed in each independent experiment (a total of 200 cells) and at least 20 random fields from three independent experiments were analyzed for each group (a total of 600 cells). Under experimental conditions in this study, an optical field of the ×60/1.4 NA oil-immersion objective lens includes 30-40 HeLa cells in average, with at least 30 cells possessing intact nucleus that are not at the edge of an observable field.<br><br>(2)The quantification of the numbers of cells with DIAPH1 or IQGAP1 at the cortex was performed using identical methods described above. Quantifications in (1) and (2) involve observation of a "yes" or a "no" phenotype (eg. mono-nucleate vs. binucleate, proteins localizing to the cortex vs. not localizing to the cortex) thus hundreds of cells in each independent experiment can be quantified within reasonable time.<br><br>(3) The number of blebs around the cortex, or length of long axis of the cell was determined in individual cell and >30 cells (in anaphase B) in each independent experiment were analyzed, with a total of 100 cells (in anaphase B) from three independent experiments were analyzed for each condition.<br><br>(4) The number of PLA signals around the cortex, the ratios of PLA signals between the two poles, the ratio of protein fluorescence intensity or blebs between two poles were determined in individual cell and >15 cells in each independent experiments were analyzed, with a total of 50 cells from three independent experiments were analyzed for each group. Quantifications in (3) and (4) involve numerical measurements of the subcellular structure (blebs), micron-scale mitotic geometry (length of long axis), individual fluorescent foci (PLA signals) or protein fluorescence intensity distribution in each individual cell thus tens of cells in each independent experiment were quantified within reasonable time.<br><br>(5) To quantify the rate of furrow ingression, a total of 5 cells in each live-cell imaging group were analyzed. To quantify the extent of cell elongation, a total of 10 cells in each live-cell imaging group were analyzed.<br><br>(6) In AFM live-cell analysis, at least 9 mitotic cells were measured in each independent experiment and 3 independent experiments were performed, with a total of at least 27 cells examined in each condition.<br><br>(7) In BLI affinity assays, each binding sequence between a ligand and an analyte was repeated in at least three independent experiments.<br><br>(8) For in vitro protein binding assays, at least three independent binding assays were performed for each condition with similar results.
Data exclusions	In all in vitro binding assays and all in vivo fixed cell analysis, no data were excluded. In live-cell imaging analysis monitoring furrow ingression or cell elongation dynamics, cells that underwent continuous metaphase-arrest within observation period (120min), or apoptosis during early anaphase, that were induced by photon-toxicity were excluded from subsequent analyses.
Replication	In all in vitro binding assays, or in vivo assays including all microscopic analysis, at least three independent experiments were perform in each condition. In BLI affinity assays each binding sequence was repeated in at least three independent experiment. In all live-cell imaging analysis, at least 5 cells over three independent experiments were analyzed. All replications were successful.
Randomization	In the binucleate phenotype analysis and DIAPH1/IQGAP1 cortical localization analysis, the selection of an observable optical field in each independent experiment was random. In these experiments, the numbers of optical fields (at least 6 fields) among each independent experiment of each group were randomized, adding up to a total of at least 20 fields in each group. In all fixed-cell analysis, live-cell imaging analysis, or AFM analysis the number of cells observed/analyzed in each independent experiment are not predetermined.
Blinding	All experiments were done with a control group set up in order to examine the effects of a certain variable (different from that of control) in that experiment thus blinding is not applicable to this study.

Reporting for specific materials, systems and methods

We require information from authors about some types of materials, experimental systems and methods used in many studies. Here, indicate whether each material, system or method listed is relevant to your study. If you are not sure if a list item applies to your research, read the appropriate section before selecting a response.

Materials & experimental systems

n/a	Involved in the study	
☐	☒	Antibodies
☐	☒	Eukaryotic cell lines
☒	☐	Palaeontology and archaeology
☒	☐	Animals and other organisms
☒	☐	Human research participants
☒	☐	Clinical data
☒	☐	Dual use research of concern

Methods

n/a	Involved in the study	
☒	☐	ChIP-seq
☒	☐	Flow cytometry
☒	☐	MRI-based neuroimaging

Antibodies

Antibodies used

anti-Maltose Binding Protein (MBP) mouse monoclonal antibody (E8032, New England Biolabs, 1:2500 dilution)
anti-His6 rabbit polyclonal antibody (MP Biomedicals, 1:500 dilution)
anti-DIAPH1 antibody (No. 610848, BD Biosciences, 1:100 dilution)
anti-IQGAP1 antibody (ab109292, Abcam, 1:250)
anti-GFP antibody (sc-390394, Santa Cruz Biotechnology, 1:500 dilution)
anti-RhoA antibody (sc-418, Santa Cruz Biotechnology, 1:200 dilution)
Rabbit anti-CLIP170 antibody (ab106524, Abcam, 1:100 dilution)
mouse anti-beta actin 4C2 (Bio-Rad MCA5775, 1:400 dilution)
mouse anti-gamma actin 2A3 (Bio-Rad MCA5776, 1:400 dilution)
goat anti-mouse antibodies conjugated to Alexa 594 (Invitrogen, 1:1000 dilution)
goat anti-mouse antibodies conjugated to Alexa 647 (Invitrogen A21241, 1:1000 dilution)
goat anti-rabbit antibodies conjugated to Alexa 647 (Invitrogen A21245, 1:1000 dilution)
goat anti-mouse antibodies conjugated to Alexa 594 (Invitrogen A11037, 1:1000 dilution)
goat anti-mouse antibodies conjugated to Alexa 488 (Invitrogen A11029, 1:1000 dilution)
anti-Aurora-A antibody (ab115883, Abcam, 1:600 dilution)
donkey anti-mouse antibody conjugated to Alexa 488 (Invitrogen A21202, 1:1000 dilution)
anti-RhoA antibody (sc-418, Santa Cruz Biotechnology, 1:500)
goat anti-mouse IgG1 antibody conjugated to Alexa-488 (Invitrogen A21121, 1:1000)
goat anti-mouse IgG2b antibody conjugated to Alexa-594 (Invitrogen A21145, 1:1000)

Validation

All antibodies are commercially available and were validated by the manufacturer as described on the associated antibody website of the manufacturer.

Eukaryotic cell lines

Policy information about cell lines

Cell line source(s)

HeLa FRT/TO Obtained from Dr. Laurence Pelletier, University of Toronto

Authentication

Cell were Zeocin and Blasticidin resistant as expected.

Mycoplasma contamination

Cell lines were tested regularly and found negative

Commonly misidentified lines
(See ICLAC register)

no commonly misidentified cells were used in the study
